# Phase Angle as a Marker of Muscular Strength in Breast Cancer Survivors

**DOI:** 10.3390/ijerph17124452

**Published:** 2020-06-21

**Authors:** Catarina N. Matias, Joana Cavaco-Silva, Mafalda Reis, Francesco Campa, Stefania Toselli, Luís Sardinha, Analiza M. Silva

**Affiliations:** 1 Exercise and Health Laboratory, CIPER, Faculdade de Motricidade Humana, Universidade de Lisboa, 1499-002 Lisboa, Portugal; cmatias@fmh.ulisboa.pt (C.N.M.); mafaldamtreis@gmail.com (M.R.); lsardinha@fmh.ulisboa.pt (L.S.); analiza@fmh.ulisboa.pt (A.M.S.); 2 Physiology and Biochemistry of Exercise Laboratory, CIPER, Faculdade de Motricidade Humana, Universidade Lisboa, 1499-002 Lisboa, Portugal; 3 ScienceCircle-Scientific and Biomedical Consulting, 1600 544 Lisboa, Portugal; jo.cvsilva@gmail.com; 4 Department for Life Quality Studies, University of Bologna, 47921 Rimini, Italy; 5 Department of Biomedical and Neuromotor Science, University of Bologna, 40126 Bologna, Italy; stefania.toselli@unibo.it

**Keywords:** body composition, breast cancer, bioimpedance, handgrip strength

## Abstract

Background: accurate prognostic tools are relevant for decision-making in cancer care. Objective measures, such as bioelectrical impedance (BI), have the potential to improve prognostic accuracy for these patients. This cross-sectional study aimed to investigate whether phase angle (PhA) derived from the electrical properties of the body tissues is a predictor of muscular strength in breast cancer survivors (BCS). Methods: a total of 41 BCS (age 54.6 ± 9.2 years) were evaluated. PhA, obtained at frequency 50 kHz, was assessed with BI spectroscopy, and muscular strength with a handgrip dynamometer. Moderate-to-vigorous physical activity (MVPA) was assessed using the International Physical Activity Questionnaire (IPAQ). Measurements were performed in the morning after an overnight fast. Results: linear regression analysis showed that PhA accounted for 22% (r^2^ = 0.22) of muscular strength variance. PhA remained a borderline predictor of muscular strength variance independently of age and MVPA. Conclusions: the findings of this study suggest that PhA is a significant predictor of maximal forearm isometric strength and a potential indicator of disease-related functionality in BCS.

## 1. Introduction

Although muscular strength has received far less attention than cardiorespiratory fitness, recent studies support the hypothesis that low muscular strength in adulthood also predicts all-cause mortality, as well as mortality due to cardiovascular disease and cancer [1,2,3,4,5,6,7,8,9,10].

As most cancer patients do not engage in physical activity programs [11], muscle dysfunction—characterized by an impairment in muscle strength—may occur. Growing evidence suggests that exercise has the ability to ameliorate and/or reverse muscle dysfunction in cancer patients [11]. As shown by Christensen et al. [11], early-stage breast cancer patients increased muscle strength by 25−35% after a 17-week resistance training program. In this regard, the maximal isometric forearm strength test is a reliable and valid method to assess muscle function [12,13]. Recent studies of breast cancer survivors (BCS) show that surgical procedures and cancer cachexia, defined by severe muscle wasting, systemic inflammation, and malnutrition [11,14], are associated with muscle strength reduction and represent risk factors for all-cause mortality [11,13,14].

Lymphedema related to surgery or radiotherapy of the axillary area is a common complication of breast cancer treatment [15], characterized by the accumulation of protein-rich extracellular fluid resulting from damaged or blocked vessels [13], and leading to a significant increase in the volume of the affected limb that results from an impairment in the ability of the lymphatic system to drain the proteins and macromolecules of the interstitium [15]. This edema formation, resulting from the fluid redistribution between extracellular water (ECW) and intracellular water (ICW) spaces, can compromise all cell functions [16,17]. Hence, an ECW/ICW ratio seems to be of great help in detecting the early onset of lymphedema. This ratio, easily obtained with bioelectrical impedance analysis, is a validated method and appears to have equal or better sensitivity than other techniques for detecting lymphedema [18].

Recently, another simple measure obtained from bioelectrical impedance analysis has received far more attention: the phase angle (PhA), which is a noninvasive simple measure directly retrieved from resistance (R) and reactance (Xc) raw data [16]. From a biophysical point of view, PhA is calculated from the arctangent of the ratio between the R and Xc, where R arises from ECW and ICW distribution, and, conversely, Xc arises from the cell membrane’s ability to take an electric load and liberate it at a later moment, after a brief delay. Therefore, it could be compared to a vessel-capacitance-like property.

Hence, PhA is considered a valuable indicator of cellular health and, as it is derived purely from electrical properties of the tissue, it avoids the typical concerns associated with prediction equations [19,20]. PhA is one of the indicators for cell membrane structure, and a lower PhA suggests decreased cellular integrity [21]. Thus PhA is considered a prognostic marker in several clinical conditions, including cancer [17], as it represents either cell death or malnutrition, both of which are characterized by changes in cellular membrane integrity. Due to this characteristic, PhA seems to be a useful predictor of impaired muscle function [16,17,22].

Gupta et al. [17] reported that PhA is a strong predictor of survival in breast cancer patients after controlling for confounders, such as stage at diagnosis and prior treatment history. Additionally, an association seems to exist between PhA and muscle strength in cancer patients, as both represent prognostic measures [23,24].

The present study emerged from the unmet need to develop a simple, easily applicable, and noninvasive tool to be used in the clinical setting to assess muscular strength. Although clinical studies using these parameters have been previously conducted, none have explored the relationship between both PhA and muscular strength in BCS after adjusting for potential confounders. Therefore, the aim of this study was to determine if PhA is a predictor of muscular strength in BCS after considering the mediation effect of lymphedema.

## 2. Methods

### 2.1. Participants

Forty-one BCS were recruited from Viva Mulher Viva Association and assessed in this observational, cross-sectional study developed at Hospital de São José, in Lisbon. Before providing written informed consent to participate, each participant was informed about the study’s goals and potential benefits.

All procedures were approved by the Ethics Committee (approval code: 27012016) of the Faculty of Human Kinetics of the University of Lisbon and conducted in accordance with the World Medical Association’s Declaration of Helsinki for human studies [25].

### 2.2. Inclusion/Exclusion Criteria

Inclusion criteria comprised patients who survived breast cancer and were currently in follow-up for their disease. Since breast cancer treatments cause body composition and muscular strength changes until six months after surgery, according to Gomes et al. [13], exclusion criteria included BCS who performed a recent (<6 months) mammary tissue removal surgery.

Recruited subjects could not be participating in other studies or have any kind of dependent relationship with study investigators.

### 2.3. Measurements

Before the morning visit to Hospital de São José, each participant was instructed to perform measurements after an overnight fast and to wear minimal clothing. Participants were further asked to remove all objects that could interfere with the bioelectrical impedance assessment.

### 2.4. Anamnesis

All patients answered a general health questionnaire, in which questions about the type of surgery were included.

### 2.5. Anthropometric Data

Participants’ weight and height were measured using a stadiometer with an incorporated scale (SECA, Hamburg, Germany) according to standardized procedures [26]. Body mass index (BMI) was calculated as body mass (kg) divided by the stature (m) squared. Waist circumference was measured at the top of the iliac crest according to the United States National Institute of Health protocol [27]. Hip circumference measurement was performed at the widest portion of the buttocks [28]. Waist to hip (WHR) ratio was calculated accordingly.

### 2.6. Phase Angle and the Ratio of Extra to Intracellular Water Compartments

Whole-body R and Xc data were obtained with bioelectrical impedance spectroscopy (BIS) (model 4200B, Xitron Technologies, San Diego, CA, USA), where participants adopted a supine position with their arms and legs abducted at a 45° angle, and right hand and foot dorsal surfaces were cleaned with alcohol. After a 10-min rest, four electrodes were placed on the cleaned surfaces and measurements were performed.

Data collection was performed with a 5- to 1-MHz spectrum, from which the software was programmed to perform biophysical modelling of the impedance data, fitting spectral data to a Cole–Cole cell suspension model [29]. This procedure derives a theoretical impedance at zero and infinity frequencies based on a non-linear curve fitting, and produces general Cole model terms, namely Re (resistance associated with ECW), Ri (resistance associated with ICW), Cm (cell membrane capacitance), and exponent α. The aforementioned Cole terms are automatically applied to equations derived from the Hannai mixture theory [30], and ECW and ICW are individually calculated based on the assumption that R0 represents the R of ECW and R∞ represent the R of the intracellular and extracellular fluid sum. Accordingly, the ECW/ICW ratio was calculated and the presence of lymphedema classified [31].

PhA was estimated by recording the voltage drop between the current applied and the two output sites, and measuring phase shift at frequency 50 kHz.

### 2.7. Maximal Isometric Forearm Strength Test

Maximal isometric forearm strength was assessed with a dynamometer (Jamar, Sammons Preston, Inc., Bolingbrook, IL, USA). Handgrip was measured on the right and left sides, with the dominant side selected for further analysis. The assessment protocol for maximal isometric forearm strength was conducted with the subject sitting in a straight-backed chair with their feet flat on the floor, their shoulder adducted and neutrally rotated, and their elbow flexed at 90° with the forearm in neutral position [32]. A 3-second contraction time was used to obtain the maximal isometric forearm strength reading.

### 2.8. Physical Activity

Physical activity was evaluated with the short form of the International Physical Activity Questionnaire (IPAQ).

From the data treatment obtained in the questionnaire, several variables were accounted for in scoring the domain of activity: minutes of physical activity (total), MET/minute/week, time spent in vigorous/moderate/light physical activity, and time spent sitting over the week or weekend. The IPAQ short form is a seven-item measure of four domains of activity: vigorous-intensity physical activity, moderate-intensity physical activity, walking, and sitting [33].

In the present study, physical activity was calculated as the sum of the days, hours, and minutes of vigorous-intensity and moderate-intensity physical activity (MVPA), presented in minutes [34].

### 2.9. Statistical Analysis

Sample size was calculated while considering a large (>0.15) Cohen’s ƒ2 effect size (appropriate for calculating effect size within a multiple regression model with continuous independent and dependent variables), with a 5% type I error, 80% power, and 4 predictors (independent variables: PhA, age, MVPA, and TPS as confounding variable). The estimated sample size was 40 participants.

Statistical analysis was performed using IBM SPSS Statistics for Mac OS version 22.0, 2010 (SPSS Inc., IBM Company, Chicago, IL, USA). Descriptive statistics (mean ± standard deviation) were performed for all measurements. All variables were checked for normality using the Shapiro-Wilk test. All the variables that resulted were normally distributed, with the exception of MVPA. Bivariate correlations were conducted in a preliminary analysis. A potential confounding factor in BCS is edema of the distal extremities, which may result from lymphedema and may potentially affect impedance measurement [35]. A univariate general linear model test was performed to investigate whether lymphedema had an impact on the relationship between PhA and strength. If this association was nonsignificant (*p* < 0.05), the sample was further analyzed as a whole. Multiple regression analysis was used to determine whether PhA was a significant predictor of muscular strength after adjusting for confounding variables (age, MVPA).

*p* < 0.05 was established as significant, except in the multiple regression analysis, in which a *p* < 0.1 indicated that the independent variable was a significant model predictor [36].

## 3. Results

A total of 41 BCS were included in this study. Anamnesis showed that 22 women underwent mastectomy (15 of which included axillary dissection) and 19 underwent conservative surgery (10 of which included axillary dissection). Additionally, five women had lymphedema.

Table 1 summarizes the characteristics of the study sample (*n* = 41).

A univariate general linear test was performed to investigate whether PhA was associated with muscular strength regardless of the presence or absence of lymphedema. Since the analysis of the interaction term lymphedema by PhA was nonsignificant (β = 0.006; *p* = 0.556) in explaining muscular strength, the whole sample was used to test the association of PhA as a predictor of muscular strength.

A multiple regression analysis was performed while adjusting the relationship between PhA and muscular strength for confounding variables (Table 2). PhA alone explained ~22% of muscular strength variance (β = 0.47; *p* < 0.001). After adjusting for age, PhA remains a significant predictor in the model (β = 0.32; *p* = 0.03). After adjusting for age and MVPA, PhA remains a significant predictor in the model (β = 0.30; *p* = 0.05).

**Figure 1:**
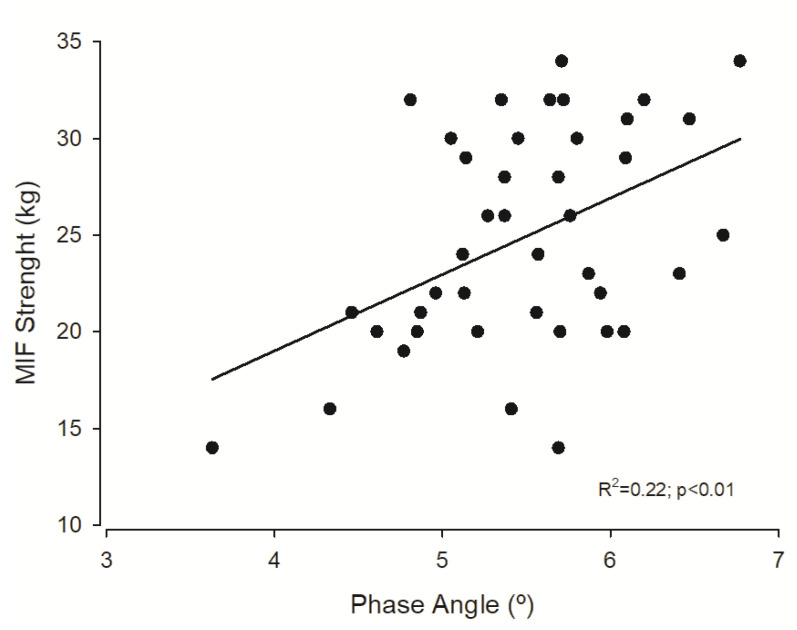
Regression plot of phase angle and maximal isometric forearm (MIF) strength.

Figure 1 represents the association between forearm muscular strength and PhA.

## 4. Discussion

This study revealed that PhA accounted for 22% of muscular strength variability, remaining a significant predictor regardless of age and MVPA. PhA represents a novel and not yet fully understood marker of cellular function [20]. A high PhA represents good cell integrity, and low PhA represents cell death or decreased cell integrity [17].

However, despite its prognostic relevance, it is still necessary to define valid cut-offs to use this marker as a clinical indicator of disease-related malnutrition in several conditions [37].

Indeed, there is an absence of consensus on the best cut-off to use, with different authors using different cut-offs according to their study population, precluding their applicability to other populations [38]. Gupta et al. [17], Hui et al. [23], and Lee et al. [39] reported longer survival in cancer patients with a PhA higher than 5.6°. In the current study, the mean PhA value (5.5 ± 0.7) may be regarded as low when considering the cut-off values presented in the above-mentioned studies. Nevertheless, all those studies were performed using single-frequency analysis equipment, while the present study used a bioelectrical impedance spectroscopy approach. With that in mind, data from single- and multi-frequency devices should not be used interchangeably, as previously noted, since a lack of agreement exists between devices in determining individual R, Xc, Z, and PhA values, with methodological and biological factors pointed out as potential justifications for the observed differences [40].

Gupta et al. [17] took a step further and established that breast cancer patients with PhA <5.6° had a median survival of 23.1 months, while those with PhA >5.6° had a median survival of 49.9 months. Importantly, the aforementioned study was conducted in cancer patients, while the present investigation only included breast cancer survivors. Although the PhA values of our sample were <5.6°, the higher mean age of our sample and methodological differences between impedance instruments may help explain the lower than expected PhA values.

PhA is also one of the best indicators of cell membrane function related to the ECW/ICW ratio [19,20,41]. Significant alterations in body fluid hydration, fluid distribution, and the ECW/body cell mass (BCM) ratio caused by certain medical conditions can affect impedance measurement and are probably associated with PhA changes [42]. According to Schwenk et al. [43], a low PhA corresponds to a high ECW/ICW ratio in systemic illnesses due to ECW expansion and ICW loss. One of the most significant confounding factors is edema of the distal extremities, which may result in lymphedema [35]. In the present study, a univariate general linear model was employed to explore whether lymphedema moderated the relationship between PhA and muscular strength. Our results failed to find any significant effect, extending the findings of Gomes et al. [13] that reported similar maximal forearm strength values, regardless of the presence or absence of lymphedema.

Despite the existence or even the influence of lymphedema, loss of function and muscle strength occurs with both disease and malnutrition and is of major clinical significance [38]. Further studies using a longitudinal approach are required to investigate whether a decrease in muscle strength in BCS is mediated by lymphedema.

Data from the present investigation suggest that PhA is a predictor of maximal isometric forearm strength measured on the dominant side using the handgrip test. The gold standard for muscle strength assessment is the force exerted in a maximum voluntary contraction, with force output measured by a dynamometer [1,11,12]. This test reflects the maximum strength derived from the combined contraction of extrinsic and intrinsic hand muscles, leading to hand joint flexion [44]. The Jamar dynamometer has been reported as the most reliable, valid, fast, and easy-to-apply instrument, with the highest calibration accuracy for maximal isometric forearm strength measurement. This tool has been recently validated in advanced cancer patients [45,46,47,48,49].

In a study conducted by Norman et al. [24], a significant association was found between PhA and forearm muscular strength. Another study [23] conducted in advanced cancer patients found that PhA was a significant predictor of survival and that lower forearm muscular strength demonstrated a trend towards shorter survival. A systematic review conducted by Neil-Sztramko et al. [50] pooled grip-strength data from 26 studies of breast cancer and reported a 22.8 kg mean value (95% CI 20.6−25.1). However, stratifying by age groups, the mean muscular strength for the 50−59-year age group was 27.7 kg. Considering that the mean age of the present BCS cohort is within this age group, the mean forearm muscular strength retrieved (24.9 kg) was below the expected. This emphasizes the need to monitor physical activity after treatment (as well as during) to help health professionals identify functional declines and promote functional outcome improvements in these patients.

Physical activity has been used as a therapeutic auxiliary in the treatment of various pathophysiological conditions [51,52]. In healthy populations, a muscular endurance decrease was found after a short period of physical inactivity [53]. In cancer patients, physical inactivity has more exacerbated consequences [54,55,56], as moderate-intensity exercise can provide a sufficient physiological stimulus to improve muscular strength in cancer survivors. One finding from the present study is that most BCS do not sufficiently engage in physical activity programs (moderate-to-vigorous physical activity (MVPA): 99.39 ± 118.67 min), not meeting minimal recommendations for weekly physical activity (150 min). Besides the cancer treatment itself, this fact may be one of the reasons for muscular strength loss. 

Despite the encouraging findings of this study, some limitations must be addressed. Firstly, the study sample is too small (*n* = 41) to detect a small-to-moderate effect size, and hence the power to detect an association may have been compromised. Secondly, the sample was not age balanced, with the youngest participant being 36 years old and the oldest 76 years old. This necessarily influences body composition parameters assessed by bioelectrical impedance equipment, such as PhA, as age is an important PhA determinant (lower PhA values are observed in older people due to a reactance reduction that parallels muscle mass loss) [57,58]. Considering the above, the authors adjusted the analysis for age as a potential confounder of the association between PhA and muscular strength. Finally, given the study’s cross-sectional nature, a causal-effect relationship could not be established, and therefore a reverse causality should not be discarded. In addition, future studies should consider evaluating changes in the ECW/ICW ratio using the segmental bioimpedance analysis approach by assessing the PhA measured in the arms.

## 5. Conclusions

The findings of this study highlight phase angle as a predictor of maximal isometric forearm strength, regardless of age and level of physical activity, suggesting its usefulness as a clinical indicator of disease-related functionality in breast cancer survivors. PhA represents a novel and not-yet-fully-understood marker of cellular function, but despite its prognostic relevance, it is still necessary to define valid cut-offs to use this marker as a clinical indicator.

## Figures and Tables

**Table 1:** Participant characteristics (*N* = 41).

Characteristic	Mean ± Std. Deviation
Age (y)	54.6 ± 9.2
Body mass (kg)	68.0 ± 11.7
Stature (cm)	159.9 ± 6.7
BMI (kg/m^2^)	26.6 ± 4.6
Waist circumference (cm)	87.1 ± 10.9
Hip circumference (cm)	100.3 ± 8.9
WHR	0.9 ± 0.1
PhA (°)	5.5 ± 0.7
ECW (L)	13.9 ± 1.7
ICW (L)	16.1 ± 3.0
E/I ratio	0.87 ± 0.09
MIF strength (kg)	25.0 ± 5.5
MVPA (min/week)	44.4 ± 49.7

Abbreviations: BMI: body mass index; WHR: waist hip ratio; PhA: phase angle; ECW: extracellular water; ICW: intracellular water; E/I ratio: ratio of extracellular and intracellular water; MIF strength: maximal isometric forearm strength; MVPA: moderate-to-vigorous physical activity.

**Table 2:** Unadjusted and adjusted models using phase angle (PhA) as the independent variable for determining maximal isometric forearm strength.

Model	Unstandardized Coefficients		Standardized Coefficients	*p*-Value
	β	Std. Error	β	
Model 1 (R^2^ = 0.22; SEE = 4.9 kg)				
(Constant)	3.51	6.57		0.60
PhA (°)	3.91	1.19	0.47	0.00
Model 2 (R^2^ = 0.35; SEE = 4.53 kg)				
(Constant)	22.60	9.23		0.02
PhA (°)	2.71	1.19	0.32	0.03
Age	−0.23	0.08	−0.39	0.01
Model 3 (R^2^ = 0.36; SEE = 4.57 kg)				
(Constant)	24.39	9.77		0.02
PhA (°)	2.48	1.26	0.30	0.05
Age	−0.25	0.09	−0.42	0.01
MVPA (min/week)	0.00	0.01	0.09	0.06

Abbreviations: PhA: phase angle; MVPA: moderate-to-vigorous physical activity.
